# Digitalization of colourimetric sensor arrays for volatile fatty acid detection in anaerobic digestion

**DOI:** 10.1016/j.mex.2019.11.002

**Published:** 2019-11-09

**Authors:** Jacob J. Lamb, Kristian M. Lien, Dag Roar Hjelme

**Affiliations:** a Department of Electronic Systems & ENERSENSE, NTNU, Trondheim, Norway; b Department of Energy and Process Engineering & ENERSENSE, NTNU, Trondheim, Norway

**Keywords:** Digitalization of colourimetric sensor arrays, Colourimetric sensor, Volatile fatty acids, Anaerobic digestion

## Abstract

During the process of converting the organic matter into methane, many volatile fatty acids (VFAs) are produced during acidogenesis and acetogenesis phases of the process. The main VFAs of interest are acetic acid, butyric acid and propionic acid. Although the production of these VFAs are essential for the production of methane, they also play an inhibitory role for many of the organisms involved in the production of biogas. As a consequence, the levels of VFAs produced in an anaerobic digester must be monitored. Current methodologies for VFA monitoring are either unspecific, or costly. Therefore, the development of a sensor method that is specific to the different VFAs, while maintaining a low cost, will facilitate the lowering of biogas production, as well as avoiding the costly biological collapse of the whole biogas production process. Here, an array of coloured dyes (colourimetric array) has been assessed for their ability to detect low concentrations of VFAs within the digestate during biogas production. This methodology lays the foundation for the development of a sensor system for use in biogas plants and could also be expanded to detect many other parameters within the biogas production process.

• Easy to establish.
• Low user input.
• Accurate measurement.

## Graphical abstract

**Figure:**
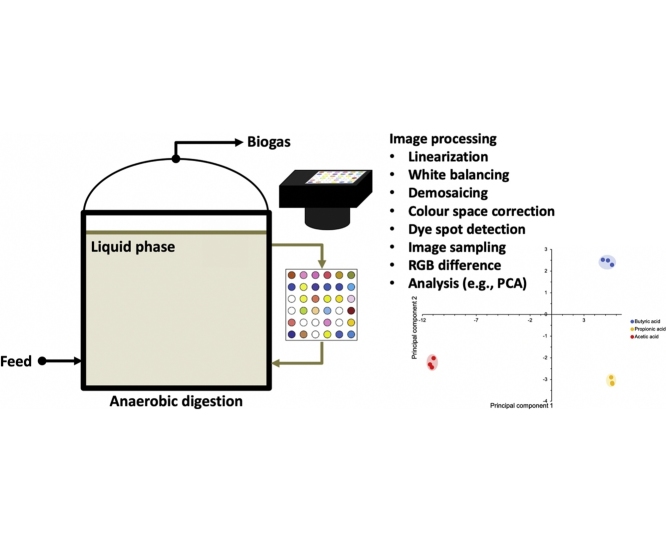

**Specification Table**

**Table:** 

Subject Area:	*Agricultural and Biological Sciences*
More specific subject area:	*Anaerobic digestion*
Method name:	Digitalization of colourimetric sensor arrays
Name and reference of original method:	*NA*
Resource availability:	*NA*

## Method details

Chemical sensors appear to be ideally suited for bioprocess monitoring compared to more complex on-line analysis techniques. With regard to VFA detection they are low cost, require relatively simple instrumentation and minimal sample preparation, and are straightforwardly integrated with control systems. Available sensor technologies for on-line monitoring of chemical variables include electrochemical, electronic, and optical technologies [1]; however, their use in bioreactors may be severely challenged by limited selectivity, repeatability, robustness, and stability [2]. In general, there can frequently be a trade-off between sensitivity and robustness when developing new sensor technologies.

One path around this challenge is to apply artificial tongue technologies based on optical sensor arrays [1]. For optical sensors, the analyte interacts with the sensor material, resulting in changing the sensor materials optical properties. The sensor array is a combination of a variety of dyes, each with different specificities for different analytes. Light, in turn, probes the sensing material. A wide range of optical techniques, in various regions of the electromagnetic spectrum, are available for probing, e.g., by refraction, scattering, reflection, absorption, and fluorescence. Probing of multiple properties can be used to enhance the sensor performance. Thus, artificial tongues based on optical sensor arrays can utilize a broad range of molecular specificities. Disposable sensor patches can be used as the chemical indicator-containing matrix, allowing a straightforward modular setup of the sensor. These can be placed directly into the anaerobic digester, possibly behind a small window, allowing an external optical component to analyse the chemical indicators. Successful application of optical tongues to metal ions, food and beverages, amino acids, proteins, bacteria, cancer, and disease diagnostic have been reviewed [3], and the methodologies for optical tongues have recently been demonstrated for use in biogas production monitoring [4]. Table 1 gives an overview of VFA detection technologies for anaerobic digestion and compares these with colourimetric sensor arrays.

**Table 1:** Overview and economic analysis of VFA detection technologies used in anaerobic digestion [4].

Sensing technology	Accuracy	VFA distinction	Sample pre-processing	Human expertise	Post-sensor data computation	Overall analysis duration (minute)	Initial Cost (USD)	Ongoing costs^a^ (USD)	Addi-tionnal varable detection	TRL^b^
Gas chromatography	5	5	5	5	2	60	30,000	300	2	6
Titrimetry	3	1	3	3	2	30	15,000	100	1	9
Infrared spectroscopy	4	5	5	5	5	60	50,000	100	3	4
Colourimetric sensors	3	5	2	3	3	2	2,000	100	5	2

Each technology is ranked from 1 (low) to 5 (high) for each category unless otherwise stated.

a Rough estimate of consumable costs per year.

b Technology readiness level.

## Colourimetric sensor array

The concept of a colourimetric sensor array is based on utilization of a matrix embedded indicator. The indicator contains either a fluorophore (fluorophore absorbs light energy of a specific wavelength and re-emits light at a longer wavelength) or a chromophore (molecules that serve to capture or detect light energy, where the chromophore is the part that causes a conformational change of the molecule when hit by light). When the analyte interacts with the immobilized indicator, the indicators optical properties (e.g., absorption, reflection, photoluminescence) change. Colourimetric changes can be detected by illuminating the whole sensor array while an imaging unit (RGB digital camera will suffice in most situations) is mounted above, enabling digital imaging of the sensor array.

A difference map can be generated by determining the colour change between images before and after the chemical of interest is present. The RGB values of the indicator dyes are then subtracted pixel by pixel to determine the difference between the dye colour before and after, yielding data for further quantitative and statistical analysis.

## Design of colourimetric sensor arrays

The choice of dyes to incorporate in a colourimetric sensor array is dependent on the application, and the dyes’ sensitivity to a range of analytes or for a more specific group of analytes. The different dye classes include; Lewis acid-base dyes, Brønsted acid-base dyes (i.e., pH indicator dyes), large permanent dipole dyes for local polarity detection, and hydrogen bonding (i.e., solvatochromic, vapochromic, or zwitterionic dyes), redox-responsive dyes and chromogenic aggregative colourants [3]. The colourimetric sensor array relies on the intermolecular interactions between the indicator and the analyte. On a molecular scale, all particles exert attractive and repulsive forces on each other, and the force between the particles must be strong to form a chemical bond or a molecule. Therefore, strong interactions will result in a higher dimensionality and discriminating powers for the sensor as chemical sensing fundamentally is molecular recognition.

Although the choice of chemo-responsive dye or fluorophore is the primary factor for the functionality of the optical sensor array, the results may be influenced by the array material. Relevant properties for such material are; inertness toward gases and liquids, high surface area and resistance to a wide range of pH. There are many different solid supports for the array construction, such as acid-free paper, porous polymer membranes, cast silicon plates and silica gel on thin layer chromatography (TLC). Independent of the sensor material, the analyte must have access to the immobilized dye in order to react. The manufacturing process of such arrays may include, robotic printing, spin coating and manual deposit of the dye.

## Sensor array construction

Thin layer chromatography silicone oxide sheets (55811-20EA, Sigma-Aldrich, Germany), were used as the sensor array plates. Small amounts of the 23 dyes were spotted onto the silicon oxide matrix plate and dried in an oven at 50 °C.

## Illumination techniques

The appearance of the sensor array is also dependent on the material and the angle of the light incident upon it. Illumination of an object can be divided into two groups regarding the orientation of the source: front-lighting or back-lighting. Different attributes (e.g., shape, colour, surface defects and transparency) of the object can be highlighted by altering the angle of the source, which affects how the light is reflected. For colourimetric sensor arrays, the goal is to detect the colours of the dye spots while minimizing surface details. Therefore, the preferred angle of the source is as perpendicular as possible. Another factor regarding how the light probes the array is the use of a diffusing material. If the light has only a direct path to the array, it will produce an uneven distribution of lighter and darker spots in the image. The diffusing material softens and disperses the light providing evenly distributed light over the object.

## Imaging techniques

Imaging can be a delicate task and the quality of the image depends on many variables. A modern digital camera has many optional settings, giving the user the opportunity to optimize the camera for any given situation. The primary three variables influencing the amount of light entering the camera is the shutter speed, aperture, and ISO. It is essential to understand the relationship between these variables and how to balance them to capture a correct image of the object. An over- or under-exposed image is a result of a poor balance between the aperture, shutter and ISO, and will result in being too bright or too dark.

Given the situation of a perfect exposure of light hitting the camera sensor, the format in which the data is captured is necessary to consider. Usually, there is a choice of either JPEG or RAW format. By using a JPEG file, the quality of the image is compromised as the camera processes and compresses the data from the camera sensor (CMOS or CCD). On the other hand, using a RAW file, the data is not compressed or processed, thus, saving as much data from the image sensor as possible. Using RAW data is valuable as it preserves the dynamic range. The dynamic range is a measurement of the light intensities captured and ranges from the darkest shade to the brightest shade of grey in the image. Therefore, a broad dynamic range will span more light intensities and give more colour tones in the image. This is valuable for a colourimetric sensor array as it will preserve the dimensionality. Unlike JPEG file, a raw file needs post-processing with computer software to gain a colour image. This is a massive advantage as the user gains full control over all the steps of the conversion. Furthermore, it is not restricted to the bit depth of 8 bit/channel. Thus, the image gain more tonal information per pixel, achieving a higher dimensionality for the colourimetric sensor array.

A straight-forward imaging rig can be constructed for optimal imaging of sensor arrays. A digital camera is ideal for use as the imaging unit, and standard halogen lights can be used for illumination. The camera must be set to a manual mode with an ISO speed of 100, white balance set to Tungsten, focus set manually, and all images saved as RAW format.

## Digital image processing

The continuous flow of digital image processing (DIP) can be divided into three levels; low-, mid- and high- level processing, where the difference is characterized by the input and output of these processes. The low-level includes techniques of filtering, contrast enhancement, and image sharpening and is considered as image pre-processing, where both the input and output are images. The mid-level extracts attribute from the image through morphological operations and segmentation. The high-level uses the at- tributes to perform image analysis and other functions related to computer vision to gain an understanding of the outcome.

DIP with regards to colourimetric sensor arrays is to calculate the colour difference between two images to produce a difference map. This is achieved through simple image subtraction. However, in addition to the difference map, it must also provide the average value of 100 pixels of the centre of every dye spot. A natural way to achieve this is to detect all the dye spots in the image and gain a set of centre coordinates of all the dye spots. This implies a more comprehensive approach involving low- and mid-level image processing.

**Fig. 1:**
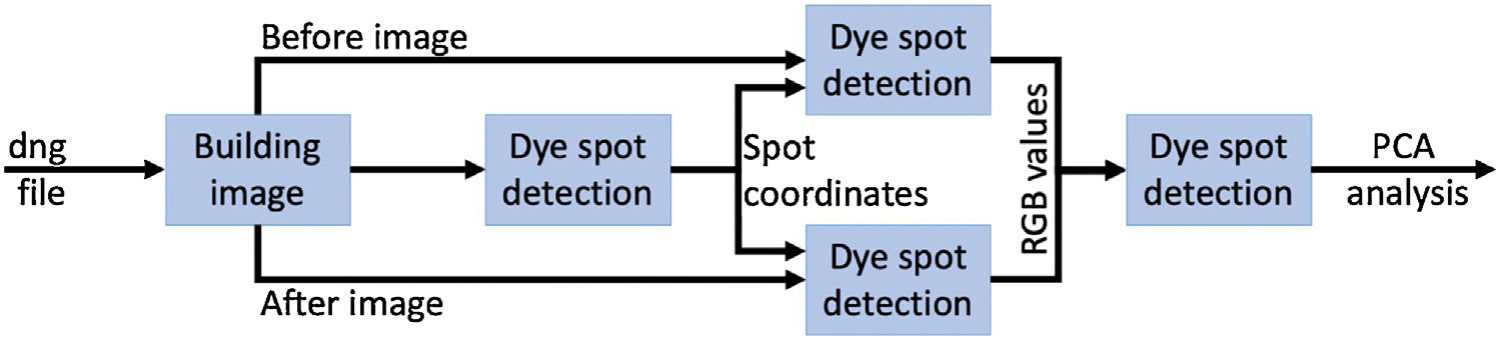
Schematic showing the processing stages of the MATLAB program from the RAW DNG image file to produce a difference map and RGB coordinate difference data for further analysis.

A program was developed within MATLAB to process the images taken by the camera to give RGB difference coordinates between before and after addition of a specific VFA. The RAW image format may also be shown as a CR2 format, depending on the digital camera in use. This was first converted into a DNG file using the Adobe DNG converter in an uncompressed format before further processing using MATLAB (Fig. 1).

**Fig. 2:**
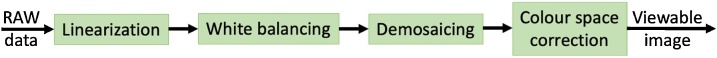
Processing steps for image building from a RAW format image to a viewable CR2 format image.

The image building phase of the process uses the CR2 image file and applies linearization, white balancing, demosaicing and colour space correction to the image file [5]. This is shown in Fig. 2. The next phase establishes the locations of all dye spots on the image to gain a set of special (x, y) coordinates. These coordinates are then used on the before and after images to automatically measure colour differences between the dye spots in response to the variable of interest (VFAs). A sampling phase calculates the average RGB values for a 10 by 10-pixel square in the centre of the dye spot. This phase results in 2 arrays of 69 data values (23 dyes by 3 colour channels), one from the before VFA addition image, and one from the after VFA addition image. The RGB difference is determined by subtracting the before VFA addition data values from their corresponding after VFA addition data values. This results in one array of 69 difference values (23 dyes by 3 colour channels).

### Linearization

Some cameras apply a non-linear transformation of the sensor data. A scaling of the sensor data is needed as there may be an offset or arbitrary scaling of the data. Furthermore, due to image noise, pixels with a value larger or smaller than the theoretical may exist. The equation bellow describes the linearization, where the RAW is the raw data and Black and Saturation is the measure of the minimal and maximal amount grey level respectively.

$$Linearized\;image=\;\left[\frac{RAW-Black}{Saturation\;-Black}\right]$$

The values are then normalized between [0–1] while remapping the errors stemming from potential noise. The image is now a CFA pattern image with only one colour channel value at each pixel location (R, G or B).

### White balancing

Every pixel in the image is still only a measured value from the capacitor in the CMOS sensor. To obtain a correct chromaticity for each channel in the CFA image, a process of white balancing must be performed. This process involves multiplication of a white-balance factor which is a specific value for each of the three channels. The white-balance factor is a vector of three values ([R, G and B]) and can be found in the meta-data. The camera used here was a Canon EOS 750D camera, which uses a RGGB CFA pattern, therefore, the white-balance factors are arranged in an array of the same size and pattern as the CFA image. This ensures that every pixel in the CFA image is multiplied by the correct white-balance factor. The resulting image is still a grey-scale image as the pixels only represents one colour channel (R, G or B).

### Demosaicing

To gain a colour image, each pixel location needs an RGB triplet. This process is referred to as demosaicing and involves interpolation of the missing channels. MATLAB has a built-in demosaicing function that uses gradient-corrected linear interpolation. The image is now an RGB image; however, it is not a correct image as colour space used is not what the monitor expects.

### Colour space correction

To obtain an accurate colour image, a conversion from the camera’s colour space to the sRGB space must be performed. This is achieved by applying a 3 × 3 matrix transformation. The process involves the multiplication of two transformation matrices, one from the camera to the XYZ colour space and one from the XYZ to the sRGB colour space. However, these matrices are opposite to the direction of the transformation; therefore, the operation must be inverted:

$$cam2rgb={\left[xyz2cam\;\times \;rgb2xyz\right]}^{-1}$$

The resulting cam2rgb transformation matrix is a 3 × 3 matrix where the columns represent the gain factor for the R, G and B channels respectively. The first row in cam2rgb is multiplied with the red colour plan, the second with the green colour plan and the third with the blue colour plan of the demosaiced image to perform the transformation.

### Dye spot detection

The primary goal of the detection is to detect all the dye spots in the before-image and gain a set of centres [x,y] coordinates of these dye spots without the need for manual input from the user. These coordinates will be the input for the next script” Sampling Images,” where a region of interest will be defined based on these coordinates. The input-image is contrast enhanced and filtered in order to analyse the image with edge detection to achieve morphological operations. The resulting image is a binary image of the input, with all the detected dyes spots.

### Sampling images

The sampling script calculates the average value of 100 pixels around the centre of each dye spot in the image. This is achieved by creating 23 masks, one for each dye spot, which is used to enclose 100 pixels (10 × 10 square in the centre of the dye spot). The start coordinates for the masks are based on the centre coordinates gained in the previous script (Dye spot detection). However, since a mask expands from top left to bottom right, the centre coordinate has to be calculated to align the centre of the mask to the centre of the dye spot. The generated mask is a 10 × 10 square and provides 100 pixel-coordinates where the R, G and B channel values are captured and averaged. The script is performed two times, one for the before image and one for the after image, resulting in two arrays (23 × 3), where the rows represent the dye spots, and the columns represent the average R, G and B channel values.

### Calculating RGB difference

The red, green and blue channel difference for each dye is calculated by subtracting the array of the before image from the array of the after image, resulting in a 23 × 3 difference-array. This will be the input for the principle component analysis (PCA). Due to the requirements of the PCA, the difference-array is converted to a 1 × 69 vector where the first three columns represent the R, G and B difference value for the first dye and the second three R, G and B values represents the second dye, and so on.

The difference-map shows the absolute colour difference and is created by subtracting the before image from the after image. For a better visual difference-map, it is preferable with the same radius for every dye spot and an entirely black background. This is achieved by creating a binary image-mask with 23 identical circular “holes.” The location of the “holes” in the image-mask is based on the centre coordinates gained from the” Detecting dye spots” script. The image-mask is multiplied with the difference-map and, therefore, the “holes” in the mask must be set to 1 (white) and the background to 0 (black). This will preserve the colour of the dye spot while generating a black background.

### Data analysis

Principle component analysis (PCA) allows the reduction of high-dimensional data to fewer, linearly independent components [1,6]. Assessment of the principal components can then be achieved by this method via assessment of the variance that is induced by the changes occurring within the bioreactor. Plots using the resulting set of principal components are often easier to visualize than the original dataset, but only if the original dataset is low dimensional in a statistical sense. Therefore, with PCA, classification is possible for the raw materials, batches, or the status of bioreactor [7].

With the RGB difference data obtained in the MATLAB program flow developed, PCA was performed using the program Minitab. The data was imported to achieve a 69 × 6 data array, in which there were 69 variables (23 dyes each having an RGB difference), from 6 difference individuals (acetic, propionic and butyric acids in high and low concentrations as shown in Table 2). The PCA within the Minitab program calculates the principle component values for each individual. An Eigenvalue is calculated for each component of the analysis, and these are plotted in a Scree plot. This shows that the first two components usually contain majority of the differentiation data and are all that is required to be displayed on a Score plot to visualise the difference between the individuals.

**Fig. 3:**
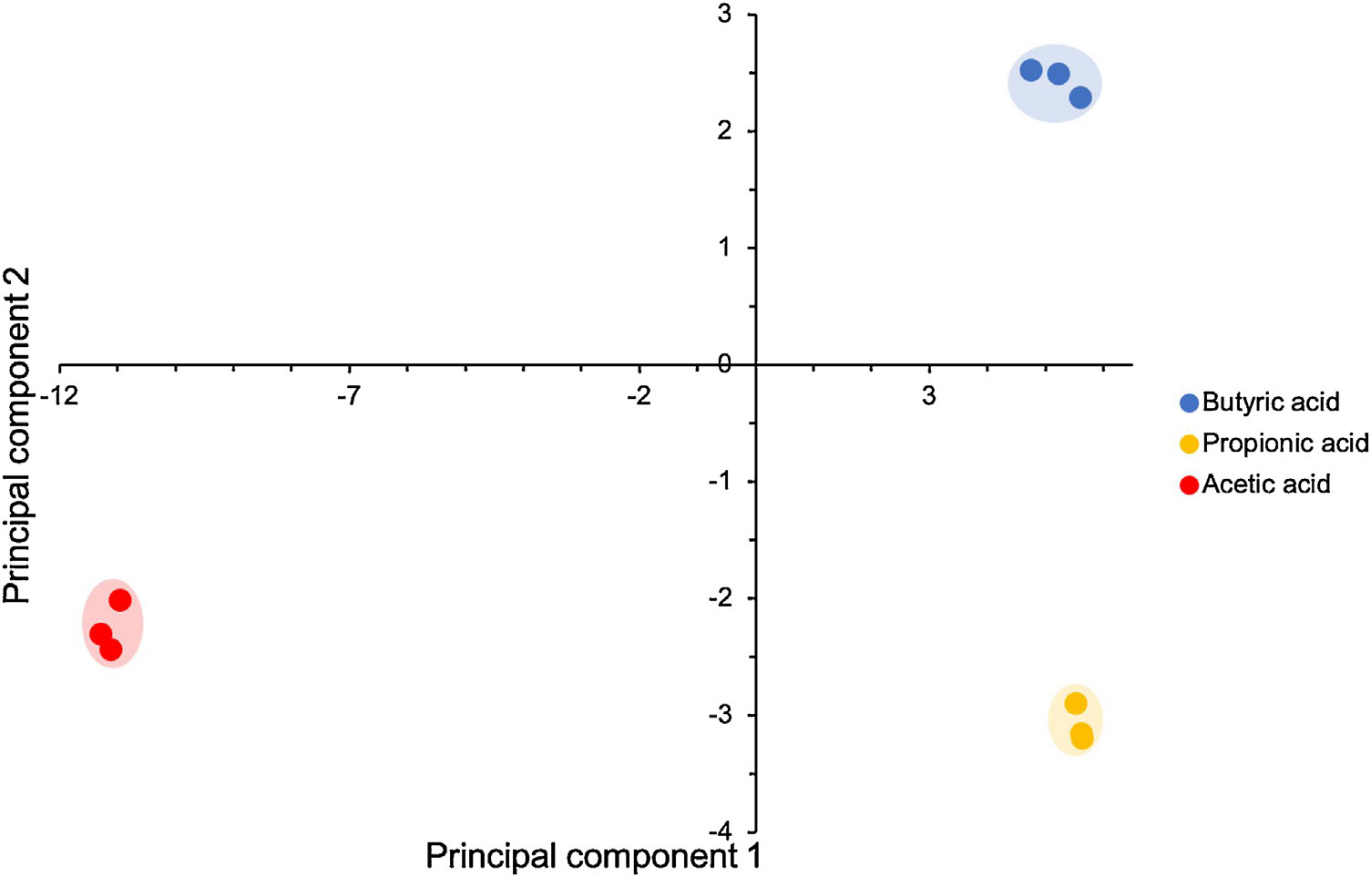
A score plot of the PCA of a 23-dye colourimetric sensor array in response to three VFAs [4].

An experiment with a 23-dye colourimetric sensor array was performed using digestate from a manure-fed anaerobic digestor. This digestate was divided into three separate vessels that were kept anaerobic. Each vessel was spiked with either acetic, propionic or butyric acid to final concentrations of 10, 5 and 0.3 mM, respectively. After exposure of the sensor array to the digestate of a specific vessel, the array was imaged. A PCA analysis of the arrays for each vessel were analysed and a PCA of the data was performed. As shown in Fig. 3 (Score plot), the sensor arrays could differentiate between the 3 VFAs when using PCA.
